# Effects of Silver Nanoparticle Exposure on Germination and Early Growth of Eleven Wetland Plants

**DOI:** 10.1371/journal.pone.0047674

**Published:** 2012-10-16

**Authors:** Liyan Yin, Benjamin P. Colman, Bonnie M. McGill, Justin P. Wright, Emily S. Bernhardt

**Affiliations:** 1 Key Laboratory of Aquatic Botany and Watershed Ecology, Wuhan Botanical Garden, Chinese Academy of Sciences, Wuhan, People’s Republic of China; 2 Center for the Environmental Implications of Nanotechnology, Duke University, Durham, North Carolina, United States of America; 3 Department of Biology, Duke University, Durham, North Carolina, United States of America; Argonne National Laboratory, United States of America

## Abstract

The increasing commercial production of engineered nanoparticles (ENPs) has led to concerns over the potential adverse impacts of these ENPs on biota in natural environments. Silver nanoparticles (AgNPs) are one of the most widely used ENPs and are expected to enter natural ecosystems. Here we examined the effects of AgNPs on germination and growth of eleven species of common wetland plants. We examined plant responses to AgNP exposure in simple pure culture experiments (direct exposure) and for seeds planted in homogenized field soils in a greenhouse experiment (soil exposure). We compared the effects of two AgNPs–20-nm polyvinylpyrrolidine-coated silver nanoparticles (PVP-AgNPs) and 6-nm gum arabic coated silver nanoparticles (GA-AgNPs)–to the effects of AgNO_3_ exposure added at equivalent Ag concentrations (1, 10 or 40 mg Ag L^−1^). In the direct exposure experiments, PVP-AgNP had no effect on germination while 40 mg Ag L^−1^ GA-AgNP exposure significantly reduced the germination rate of three species and enhanced the germination rate of one species. In contrast, 40 mg Ag L^−1^ AgNO_3_ enhanced the germination rate of five species. In general root growth was much more affected by Ag exposure than was leaf growth. The magnitude of inhibition was always greater for GA-AgNPs than for AgNO_3_ and PVP-AgNPs. In the soil exposure experiment, germination effects were less pronounced. The plant growth response differed by taxa with *Lolium multiflorum* growing more rapidly under both AgNO_3_ and GA-AgNP exposures and all other taxa having significantly reduced growth under GA-AgNP exposure. AgNO_3_ did not reduce the growth of any species while PVP-AgNPs significantly inhibited the growth of only one species. Our findings suggest important new avenues of research for understanding the fate and transport of NPs in natural media, the interactions between NPs and plants, and indirect and direct effects of NPs in mixed plant communities.

## Introduction

With the rapid growth of nanotechnology, there are growing concerns over the potential adverse impacts of engineered nanoparticles (ENPs) in the environment. However, our understanding of how ENPs may affect organisms within natural ecosystems, lags far behind our rapidly increasing ability to engineer novel nanomaterials [1]. To date, research on the biological impacts of ENPs has primarily consisted of controlled lab studies of model organisms with single species in culture media [1], [2]. These types of highly controlled experiments are essential for elucidating the mechanisms of ENPs toxicity; however, pure culture research is rarely sufficient to predict the impacts of a potential contaminant in the complex soils, sediments, or waters found in natural environments. Numerous authors have reported that Ag nanoparticles (AgNPs) are toxic to microbes in pure culture studies [3], but several studies have shown AgNPs to have no effect on microbial community composition or activity in natural sediments [4], [5]–suggesting that physicochemical interactions in the environment may significantly dampen ENP toxicity. Our limited understanding of how the physicochemical interactions between soils and ENPs will affect the chemistry and behavior of AgNPs limits our ability to extrapolate the effects of pure culture research to realistic field scenarios.

Silver nanoparticles are one of the most widely used ENPs in consumer products where they are increasingly used for their antimicrobial properties [6]. In addition, they have been shown in pure culture studies to be toxic to animal and human cells [7], [8], algae [9], [10] and fish [11], [12]. To date, there have been only a few reported studies of the impact of AgNPs on vascular plants, but these have consistently shown that AgNPs have detrimental effects on plant growth [13]–[16]. Stampoulis et al. [13] reported that 100 nm AgNPs at 100 and 500 mg/L resulted in 41% and 57% decreases in the biomass and transpiration rates, respectively, of *Cucurbita pepo* (zucchini), as compared to control plants. Kumari et al. [14] found AgNPs had cytotoxic and genotoxic impacts on *Allium cepa* root tip cells. Gubbins et al. [15] reported that AgNPs could inhibit the growth of *Lemna minor*. Jiang et al. [16] reported that AgNPs significantly decreased plant biomass, plant tissue nitrate-nitrogen content, chlorophyll a/b and chlorophyll fluorescence (Fv/Fm) in an aquatic macrophyte (*Spirodela polyrhiza,* greater duckweed). In each of these studies plant toxicity was only tested in pure culture.

In our own previous research, we exposed seeds of the annual ryegrass *Lolium multiflorum* to a range of exposure concentrations of dissolved Ag (AgNO_3_) and 6 nm gum Arabic coated AgNPs. Seeds exposed to AgNPs had severely stunted root growth and we were able to conclusively demonstrate that the toxicity of AgNPs exceeded that of identical doses of dissolved Ag (as AgNO_3_) indicating that cell damage could be directly attributed to the nanoparticles themselves [17]. These experiments were performed in pure culture. In the current study, we were interested in expanding upon these findings by examining: whether the toxicity responses we observed were consistent across a variety of wetland plant species; and whether the effects observed in pure culture would affect seeds germinated in soil. For this study we exposed 11 species of wetland plants to two types of AgNPs as well as AgNO_3_ under pure culture conditions and after planting in natural wetland soils. We asked: (1) Are the effects of AgNPs of equal or greater magnitude than the effect of dissolved Ag on the germination and growth of each plant species? (2) How do plant species differ in their response to AgNPs and dissolved Ag exposure? and (3) Are the effects observed in pure culture consistent in direction or magnitude with the effects observed in natural soils?.

## Materials and Methods

### Ag Suspensions

PVP-coated Ag nanoparticles (PVP-AgNPs) were purchased as a dry powder (Nanoamorphous Materials, Los Alamos, USA), and suspended in deionized water (resistivity >18 MΩ cm; suspension pH = 5.8) to make a 250 mg Ag L^−1^ stock suspension by sonicating them for 10 minutes using a probe type sonicator (Misonix, QSonica LLC, Newton, USA). GA-AgNPs stock suspension of 250 mg Ag L^−1^ used here were from the same batch as we used in our previous study [17]. AgNO_3_ was used to compare the toxicity of AgNPs and dissolved Ag.

### Physicochemical Characterization of the AgNP Stock Suspensions

Both GA-AgNPs and PVP-AgNPs used were roughly spherical. The size distributions obtained by transmission electron microscopy were 6.0±1.7 and 21.0±17.0 nm for the GA-AgNPs and PVP-AgNPs, respectively (Table S1 and Figure S1). In their stock suspensions, the two AgNPs have negative surface charges with a zeta potential of −49.5±1.5 mV and −22.5±1.4 for the GA-AgNPs and PVP-AgNPs, respectively (Table S1). Detailed information of GA-AgNPs synthesis and analysis have been previously published [17]. Detailed information on particle characterization of PVP-AgNPs can be found in Meyer et al. [18].

### Germination in Pure Culture

Eleven species of wetland plants (*L. multiflorum, Panicum virgatum, Carex lurida, C. scoparia, C. vulpinoidea, C. crinita, Eupatorium fistulosum, Phytolacca americana, Scirpus cyperinus, Lobelia cardinalis, Juncus effusus*) were chosen to represent a taxonomically and functionally diverse set of species (Table 1). All seeds were purchased from Ernst Conservation Seeds (Meadville, PA, USA) and kept in the dark at 4°C until use.

**Table 1:** Change in germination rate, leaf length, and root length in the pure culture experiment.

Growthform	Family	Species	Seed Mass(mg)	Change in germination			Change in leaf length			Change in root length		
				PVP	GA	AgNO_3_	PVP	GA	AgNO_3_	PVP	GA	AgNO_3_
Monocot	Cyperaceae	*Carex lurida*	2.36	−0.16	0.04	−0.06	*0.34*	−**0.22**	0.09	*0.47*	−**0.89**	−**0.63**
Monocot	Cyperaceae	*Carex crinita*	0.63	−0.04	−0.16	0.18	0.05	−**0.34**	−**0.29**	−**0.31**	−**0.82**	−**0.95**
Monocot	Cyperaceae	*Carex scoparia*	0.38	0	−0.1	*0.21*	−0.04	−**0.31**	−0.05	−**0.44**	−**0.96**	−**0.76**
Monocot	Cyperaceae	*Carex vulpinoidea*	0.35	−0.17	−0.29	0.03	−0.12	−**0.23**	−**0.2**	−**0.59**	−**0.92**	−**0.71**
Monocot	Cyperaceae	*Scirpus* *cyperinus*	0.01	0.08	−**0.71**	0.09	0.87	−**0.22**	−0.08	−**0.46**	−**0.93**	−**0.87**
Monocot	Juncaceae	*Juncus effusus*	0.01	0.18	−**0.82**	*0.71*	−0.07	−0.14	−0.12	−	−	−
Monocot	Poaceae	*Lolium multiflorum*	2.09	−0.09	−0.18	−0.19	−**0.25**	−**0.55**	−**0.45**	−**0.33**	−**0.9**	−**0.43**
Monocot	Poaceae	*Panicum virgatum*	1.75	0.24	−0.06	*0.79*	−0.05	−0.03	−0.01	*0.64*	−**0.82**	*0.64*
Dicot	Asteraceae	*Eupatorium fistulosum*	0.23	0.23	*0.52*	*0.69*	−0.05	−0.23	−0.25	−0.14	−**0.87**	−**0.77**
Dicot	Campanulaceae	*Lobelia cardinalis*	0.04	−0.25	−0.38	−0.38	−0.11	−0.22	−0.22	−**0.47**	−**0.61**	−**0.59**
Dicot	Phytolaccaceae	*Phytolacca americana*	7.82	0.02	−**0.28**	*0.3*	−0.1	0.07	−0.01	*1.76*	0.2	*2.55*

Numbers in the “change” columns represent fractional change when comparing the PVP-AgNPs, GA-AgNPs, and AgNO_3_ treatments with the DI control treatment. The 11 species of wetland plants chosen for the experiment including are listed including key characteristics for each species. Measurements were taken after 20 days of growth. Significant (p<0.05) *reductions* in germination or growth are indicated in bold and significant *increases* in germination or growth are indicated in italics. Data on seed mass from USDA PLANTS database (www.plants.usda.gov).

For each individual treatment ∼200 seeds of each species were soaked for 1 h in 5 mL of either 1, 10 or 40 mg Ag L-1 of either GA-AgNPs, PVP-AgNPs, AgNO_3_, or a deionized water (DI) control without surface sterilzation. We did not include a KNO_3_ N-control here since our previous study showed that NO3-N in AgNO_3_ had no effect on seed germination and seedling growth of L. multiflorum [17]. Once seeds were placed in the treatment solution, each jar was shaken for five seconds three times over the course of the hour to ensure all the seeds were thoroughly and evenly in contact with the solutions. One piece of filter paper was put into each 100 mm * 15 mm petri dish, and 4 ml of the appropriate treatment test solution was added. Seeds were then transferred onto the filter paper, with 35 seeds per dish. Petri dishes were covered and sealed with tape and randomly placed together in a greenhouse set to 25–30°C in daytime (16 h) and 15–20°C at night (8 h). Five replicates of each treatment were prepared, for a total of 50 petri dishes per plant species.

We measured seed germination rate, shoot and root length for all replicates after 20 days of incubation with the exception of *L. multiflorum,* which we measured on day 10 due to quick germination and growth rate. A seed was considered to have germinated when the radicle or plumule emerged from the seed coat and seed germination rate (GR) was calculated as the proportion of the total seeds that germinated. Shoot length (SL) and root length (RL) were measured by slide caliper. From these data we made three calculations:

1. treatment effects on germination
  (GR_TRT_ – GR_CONTROL_)_/_GR_CONTROL_
2. treatment effects on leaf length
  (SL_TRT_ – SL_CONTROL_)_/_SL_CONTROL_
3. treatment effects on root length
  (RL_TRT_ – RL_CONTROL_)_/_RL_CONTROL_

### Germination in Soil

Twenty trays (25×51×5 cm) were filled with screened soil obtained from the floodplain of Sandy Creek, a small stream directly adjacent to the Duke University campus. We saturated soils by adding 3 L of deionized water to each soil-filled tray before sowing the seeds. Each container was sown with a seed mixture of 7 species (*L. multiflorum, C. crinita, C. scoparia, C. vulpinoidea, C. lurida, Phytolacca americana, E. fistulosum*). We used data from the DI control treatment in the pure culture germination assay to determine the number of seeds for each species, with a target density of 10 seedlings per species per tray. *S. cyperinus, J. effusus, Panicum virgatum* and *L. cardinalis* were not used here due to their slow germination or prohibitively small seedling size. Immediately after the seeds were sown, we sprayed each tray with 250 mL of a 40 mg Ag L^−1^ solution as either GA-AgNPs, PVP-AgNPs, AgNO_3_, KNO_3_, or DI (0 mg Ag L^−1^). Five replicates of each treatment were prepared for a total of 25 trays. Trays were covered with a clear plastic lid to help retain moisture and randomly placed together in the same greenhouse as the petri dishes.

After five weeks, the germinants had established sufficiently to survive without the tray lids. At this time we began adding 1000 mL of DI to each tray every two days. After another two weeks of growth we destructively sampled the experiment. All aboveground biomass was cut at the soil surface, identified and sorted to the lowest possible taxonomic classification, placed into paper bags for oven drying at 80°C for 48 h and then weighed for aboveground biomass. Because the seedlings of the four *Carex spp.* were harvested before they could be distinguished at the species level we lumped all four species together as *Carex spp*. Aboveground biomass is the only response variable investigated, due to the inability to separate roots to species.

### Statistical Analysis

All errors are expressed as standard deviations (SD). Differences between treatments for the different measured variables were tested using one-way analysis of variance (ANOVA), followed by Tukey’s HSD post-hoc test when significant differences were found (p<0.05).

## Results

### AgNPs Effects on Germination and Growth in Pure Culture

Exposure to GA-AgNPs or AgNO_3_ significantly affected seed germination rates for multiple plant species, while exposure to PVP-AgNPs had no measurable effects on germination (Table 1 and S2). Germination responses differed between Ag forms and species; exposure to AgNO_3_ promoted germination rates for 5 of the 11 species, while exposure to GA-AgNPs significantly promoted the germination rate of only one species, *E. fistulosum,* while inhibiting the germination of *S. cyperinus, J. effusus* and *P. americanum* (Table 1).

All three forms of Ag affected leaf growth for some species although the direction and magnitude of the effects differed by treatment and species. Only *C. lurida* leaf growth responded positively to Ag exposure. All three Ag treatments elicited this response, however PVP-AgNPs had a significant effect only at the highest dose (40 mg L^−1^), AgNO_3_ had a significant effect only at the intermediate dose, and GA-AgNPs had a stimulatory effect at the lowest dose and an inhibitory effect at the highest dose (Tables 1 and S3). The grass *L. multiflorum* responded negatively to all three forms of Ag with significant negative effects of PVP-AgNPs and AgNO_3_ were observed at the highest dose, and significantly less leaf growth from GA-AgNP exposure at both the intermediate and highest dosing scenario (Tables 1 and S3). High dose (40 mg Ag L^−1^) exposures led to 55%, 45% and 25% reductions in leaf growth on average in the GA-AgNP, AgNO_3_ and PVP-AgNP treatments respectively. Two species, *C. scoparia* and *S. cyperinus* had significantly reduced leaf growth when exposed to 40 mg Ag/L GA-AgNPs but were unaffected by exposure to PVP-AgNPs or AgNO_3_ (Table 1). Both *C. crinita* and *C. vulpinoidea* had similar reductions in leaf growth in response to high doses of either GA-AgNPs or AgNO_3_ (Table 1).

In general, root growth was much more sensitive to Ag exposure than was leaf growth. We documented significant root growth responses to all three Ag exposure scenarios for nine of the ten plant species for which root growth occurred (Tables 1 and S4). The magnitude and direction of the effect differed by treatment and species. Root growth of the forb *E. fistulosum* did not respond to PVP-AgNP but did respond to GA-AgNPs and AgNO_3._ Two species, *Phytolacca americana* and *Panicum virgatum,* responded to Ag treatments by growing significantly longer and more curved roots compared to root growth in DI, although at the highest concentration of GA-AgNPs *Panicum virgatum* root growth was significantly inhibited (Tables 1 and S4). All other species had significantly shorter roots upon exposure to any of the three Ag treatments. The magnitude of inhibition was always greater for GA-AgNPs than AgNO_3_ or PVP-AgNPs. We documented significantly greater reductions in root growth under GA-AgNP exposure for six species, *L. multiflorum, C. lurida, C. scoparia, C. vulpinoidea, E. fistulosum*, and *S. cyperinus,* while there was no significant difference between the effect of AgNO_3_ and GA-AgNPs in root growth for two species, *C. crinata* and *L. cardinalis* (Tables 1 and S4).

### AgNPs Effects on Germination and Growth in Soil

**Figure 1:**
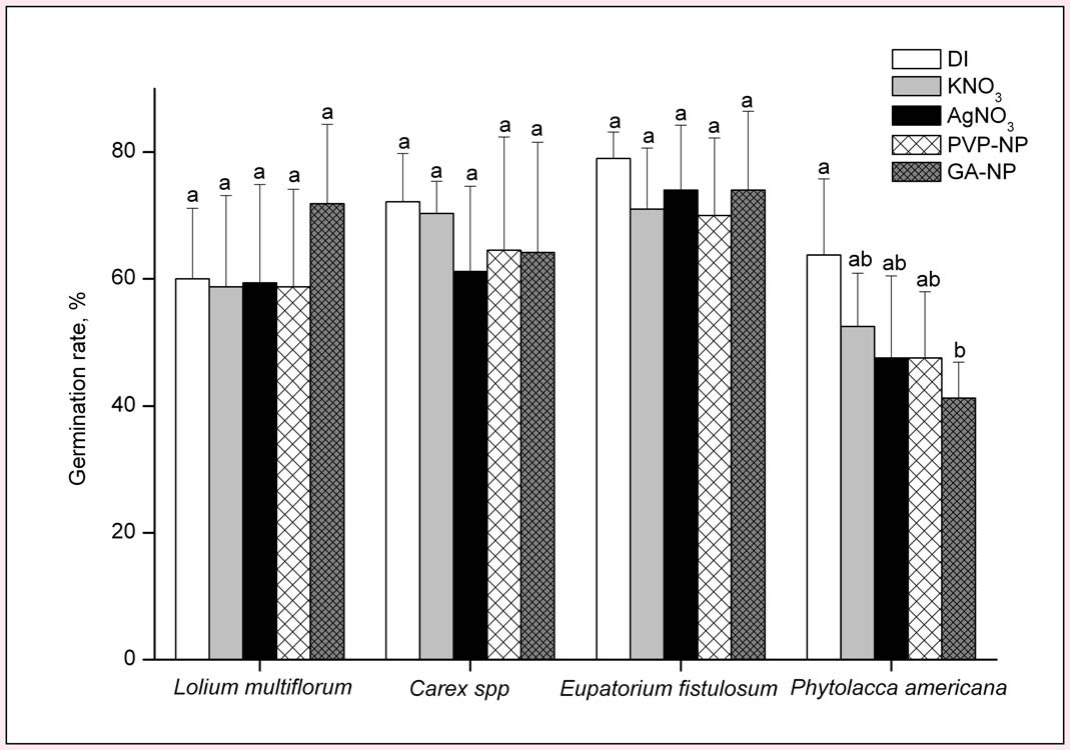
Effect of AgNPs and AgNO_3_ on the seed germination rate of wetland plants after seven weeks of silver exposure in soil. Different letters show significant differences (p<0.05).

**Figure 2:**
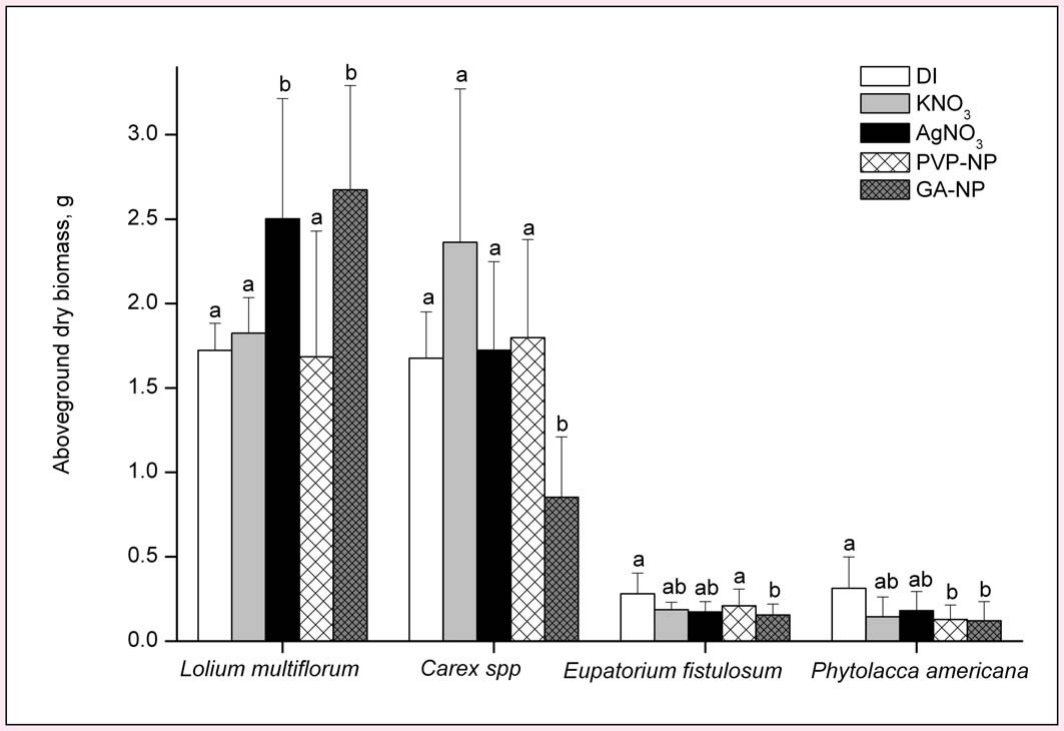
Effect of AgNPs and AgNO_3_ on the aboveground biomass of wetland plants after seven weeks of silver exposure in soil. Different letters show significant differences (p<0.05).

The only significant effect of Ag on germination in the soil exposure experiment was inhibition of germination of *Phytolacca americana* by GA-AgNPs (Figure 1). Though the effects on germination were minimal, all three forms of Ag had significant impacts on aboveground biomass. The magnitude of plant biomass change was greater for GA-AgNPs than for PVP-AgNPs or AgNO_3_. Exposure to GA-AgNPs affected seedling growth for all tested plant species while exposure to PVP-AgNPs and AgNO_3_ affected seedling growth for only one species each, *L. multiflorum* and *Phytolacca americana*, respectively (Figure 2). Seedling growth responses differed between species. The grass *L. multiflorum* responded positively to both the GA-AgNP and AgNO_3_ treatments, and its aboveground biomass increased by 55% and 45%, respectively, as compared to control plants. In general, aboveground biomass of the other species demonstrated a negative response to the Ag treatments. Both *Carex spp.* and *E. fistulosum* had significantly reduced aboveground growth when exposed to GA-AgNPs. *Phytolacca americana* aboveground growth was reduced 62% and 65% when exposed to PVP-AgNPs and GA-AgNPs, respectively.

## Discussion

Here we examined the toxicity of GA-AgNPs, PVP-AgNPs, and AgNO_3_ to the seeds of 11 species of wetland plants in pure culture and a mixture of a subset of these species in soil. We found that GA-AgNPs had effects equal to or greater in magnitude than AgNO_3_ on seedling growth, confirming that the high toxicity of AgNPs is not only due to the ionic Ag content. We also found that plant species differ in their susceptibility to AgNPs and AgNO_3_ and that AgNPs’ toxicity to wetland plants under realistic growth conditions is only partially consistent with results from pure culture experiments. These results suggest that the increasing release of AgNPs into the environment could have effects on wetland plant communities.

### AgNPs’ Effects on Plants in Pure Culture

The presence of ionic Ag has been shown to be important to the toxicity of AgNPs to human hepatoma cells [19], *Caenorhabditis elegans* [20], algae [9] and bacteria [21]. However, it was reported that the phytotoxic effect of AgNPs to *Arabidopsis thaliana* and *C. pepo* could not be fully explained by the presence of ionic Ag [13], [22]. Our previous study also showed the toxicity of GA-AgNPs to *L. multiflorum* was not due solely to the ionic Ag ions [17]. Our results here demonstrate that under direct exposure scenarios in pure culture and soil conditions, AgNO_3_ primarily stimulated seed germination, while AgNPs had either no effect (PVP-AgNPs) or primarily an inhibitory effect (GA-AgNPs). New germinants’ root lengths across six plant species were significantly reduced in the pure culture experiment when seeds were exposed to GA-AgNP versus AgNO_3_. Given the higher toxicity to both shoot and root growth of GA-AgNPs when compared to AgNO_3_, we suggest that the high toxicity of GA-AgNPs was not only due to the presence of ionic Ag. However, it is possible that interactions between the plants and the AgNPs enhanced the release of Ag from the AgNPs, as reported for algae [10], or that AgNP uptake by the plants provide a source of Ag^+^, and AgNP-cell-Ag^+^ interactions at the cell interface enhance AgNPs’ toxicity.

Particle size, coating and surface charge have been shown to greatly affect NPs’ toxicity [23]–[25]. Plant cell walls function as natural sieves, and NPs may have to penetrate cell walls and plasma membranes of epidermal layers in roots to enter vascular tissues (xylem) in order to be taken up and translocated through stems to leaves [26]. The pore size of plant cell walls is usually in the range of a few nanometers [27], which is much smaller than 21 nm PVP-AgNPs while potentially in the range of 6 nm GA-AgNPs. This may partially explain why our 6 nm GA-AgNPs have stronger effects than our 21 nm PVP-AgNPs. It is reported that GA-AgNPs are more toxic to *Nitrosomonas europaea* than PVP-AgNPs due to their different coatings [28]. It has also been reported that surface charge plays an important role in metal nanoparticle toxicity to bacteria, with more negative AgNPs being the least toxic to *Bacillus spp.* due to an electrostatic barrier which limited the cell-particle interactions [25]. However, both GA-AgNPs and PVP-AgNPs used in our experiment were negatively charged, with the GA-AgNPs are more strongly charged than PVP-AgNPs. In this experiment it is likely that the combination of size, coating and perhaps even surface charge played a role in making the 6 nm GA-AgNPs show stronger effects than the 21 nm PVP-AgNPs.

Our pure culture results showed that both shoot and root growth was much more sensitive to Ag exposure than seed germination. This is consistent with previous studies that report NPs had less of an effect on seed germination than seedling growth [29], [30]. This may be explained by the protective effect of the seed coat [31]. Since roots are the first target tissue to confront pollutants, toxic symptoms seem to appear more strongly in roots rather than in shoots [32]. Our previous study showed that most of the Ag in the the plant appeared to remain associated with its roots, and the translocation factor ([Ag] in shoots/[Ag] in roots) was very low. This could explain why the root response was stronger than the leaf response to Ag.

The magnitude and direction of plant growth responses to AgNPs and AgNO_3_ exposures differed between species. Of all species studied, only *C. lurida* responded to Ag exposures with significant increases in both leaf and root biomass, and this positive response was only observed in the PVP-AgNP exposure. Both *Phytolacca americana* and *Panicum virgatum,* also grew significantly longer roots in response to Ag treatments, in this case in response to both PVP-AgNPs and AgNO_3_ and without any measurable impact on leaf length. These observed increases in roots and leaf biomass in several taxa may appear to be in marked contrast with much of the emerging literature showing decreased growth in response to AgNP exposure[13]–[16], but is consistent with previous published reports showing sublethal stimulation of growth (i.e., hormesis) caused by AgNO_3_ [33]. Our previous study also found that plant root tips bent away from gravity in Ag treatments [17]. Given that auxin transport toward the root apex is required for gravitropism in roots [34], we speculate that Ag-induced damage may cause the loss of gravitropism in roots through disruption of auxin transport.

While three species showed an increase in growth in response to AgNP or AgNO_3_ exposure, the majority of plants had significantly shorter roots upon exposure to any of the three Ag treatments. We hypothesize that plants may cope with the Ag toxicity either by an “escape strategy” involving rapid root elongation and curving or by a “quiescence strategy” involving persistence under high Ag concentration with minimal activity, as reported for plants when they suffer other stresses such as flooding [35]. Such survival strategies do not appear to be clearly linked to taxonomy, nor do germination effects of AgNPs appear to be predicted by seed size. While we tested 11 species, our representation of the full diversity of plants is limited, thus limiting our ability to tie observed responses to plant classification. Nonetheless, it is interesting to note that none of the dicots showed significant effects on leaf growth.

### AgNP Effects on Plants in Soil

From the pure culture experiment, we clearly observed that GA-AgNPs had effects on germination and seedling growth equal to or greater in magnitude to those of AgNO_3_ and that plant species differed in their susceptibility to Ag exposure. In the soil experiment, seeds were germinated in soil to examine whether the effects observed in pure culture on germination and shoot growth could be documented when plants are grown in the chemically complex environment of soil over a longer time period.

Our results generally showed that the impacts of AgNPs on seed germination and plant growth in soil were consistent with those observed in pure culture. GA-AgNPs significantly inhibited *Phytolacca americana* seed germination and *Carex spp.* aboveground growth both in soil and pure culture. PVP-AgNPs had relatively low toxicity to plant seed germination and seedling growth both in soil and pure culture. These results suggest that there is promising consistency between AgNP toxicity observed in pure culture systems and potential AgNP toxicity in the natural environment.

However, for some species and Ag treatments, the effects of Ag on plants in soil were either attenuated when compared to pure culture, or in some cases the direction of the effect changed (i.e., stimulation instead of inhibition of growth). For example, GA-AgNPs and AgNO_3_ both significantly promoted seed germination of *E. fistulosum* in pure culture, but had no effect on *E. fistulosum* grown in soil. In contrast, *L. multiflorum* responded negatively to GA-AgNPs and AgNO_3_ in pure culture, but positively to both forms of Ag in soil. There are several possible explanations for these changes in germination and growth. First, it could be that they represent a decrease in effective concentration due to dosing AgNPs in a larger volume of soil as opposed to on a much smaller volume in a petri dish with filter paper. Second, it could be that the seeds and seedlings experienced a decreased impact of Ag due to surface modification and interaction of AgNPs and Ag^+^ with the organic and mineral phases of soil. AgNPs may aggregate or be complexed by ligands which can cause a decreased in toxicity [1], [4], [5]. Both dilution in soil and complexation/aggregation of Ag^+^ and AgNPs would lead to lower exposure to seeds and seedlings. This could both explain the lack of impact on germination in soil when compared to pure culture, as well as the increased growth of *L. multiflorum*, which could be subtoxic stimulation of growth–also observed in other species in pure culture.

However, given that in the soil experiment plants were grown in multispecies mixtures, direct effects of AgNPs and AgNO_3_ on plants were not the only potential explanations; impacts in mixed communities of plants can be due to indirect NP effects mediated by altered species interactions. For example, while the increased performance of *L. multiflorum* under GA-AgNP in soil could have been due to alleviation of toxicity from GA-AgNPs in soil, it could also represent improved growth by *L. multiflorum* due to a release from competition due to the negative impacts of GA-AgNPs on competing *Carex spp*.

In this experiment, we selected Ag exposure concentrations based primarily on previous studies of AgNPs on plant growth. In this study our intention was to explore the extent of variation in plant species responses to two different types of AgNPs relative to AgNO_3_, and under two different exposure scenarios (pure culture vs. in soil) rather than to approximate realistic doses. It is important to note that even our lowest concentrations are well above the worst case scenarios of AgNP loading to surface waters [36]). These application concentrations, however, may represent realistic doses for plants growing in wetlands that receive and accumulate chronic low level AgNP inputs. Our results suggest that there is likely to be substantial variation in both the sign and the magnitude of individual plant species responses to AgNP exposure, that the plant growth response to AgNPs can be weaker (for PVP-AgNPs) or stronger (GA-AgNPs than for AgNO_3_).

One final contrast between our pure culture and soil experiments is that toxicity of AgNO_3_ was more attenuated in soil than the toxicity of GA-AgNPs. In the pure culture experiment, both GA-AgNPs and AgNO_3_ significantly inhibited the root growth of *E. fistulosum* and four *Carex spp*. However, inhibition from GA-AgNPs in soil was still significant while no significant effect of AgNO_3_ was found. Also, the promotion effect of AgNO_3_ on seed germination found in pure culture was absent in soil. Many ligands common in soil solution (e.g, thiols, sulfide, chloride, and phosphate) can decrease the bioavailability and mitigate the toxicity of Ag at relatively low concentrations [37], [38]. While ligands can also bind and alter toxicity of AgNPs, low concentrations of chloride have been shown to mitigate Ag toxicity but not that of AgNPs [38]. Thus the greater maintenance of toxicity of GA-AgNPs than AgNO_3_ might be due to the presence of common ligands in soil that preferentially decrease the bioavailability and toxicity of Ag^+^. It is reported that under ambient conditions new smaller AgNPs can be formed in the vicinity of the parent AgNPs, which is hypothesized to be due to Ag^+^ release and subsequent AgNP formation by chemical- and/or photo-reduction [39]. The potentially entangled nature of AgNPs/Ag^+^ in the environment makes the interactions between AgNPs, Ag^+^, and plant tissues even more complicated. While investigating the form and fate of AgNPs in our experiments was beyond the scope of these experiments, our findings suggest important new avenues of research for understanding the fate and transport of NPs in natural media, the interactions between NPs and plant tissues, and indirect and direct effects of NPs in mixed communities.

## Supporting Information

Figure S1 TEM images of 6 nm GA-AgNPs (Left) and 21 nm PVP-AgNPs (right) before any incubation.
(DOC)

Table S1 Physico-chemical characterization of the AgNPs before any incubations.
(DOC)

Table S2 Effect of AgNPs and AgNO_3_ on the seed germination rate (%) of 11 species of wetland plants after 20 days of exposure.
(DOC)

Table S3 Effect of AgNPs and AgNO_3_ on the leaf length (cm) of 11 species of wetland plants after 20 days of exposure.
(DOC)

Table S4 Effect of AgNPs and AgNO_3_ on the root length (cm) of 11 species of wetland plants after 20 days of exposure.
(DOC)
