# Supplementary figures and images for: Effects of Silver Nanoparticle Exposure on Germination and Early Growth of Eleven Wetland Plants
Source: PLoS One. 2012 Oct 16;7(10):e47674. doi: 10.1371/journal.pone.0047674 (PMC3473015; doi:10.1371/journal.pone.0047674)

Figure S1


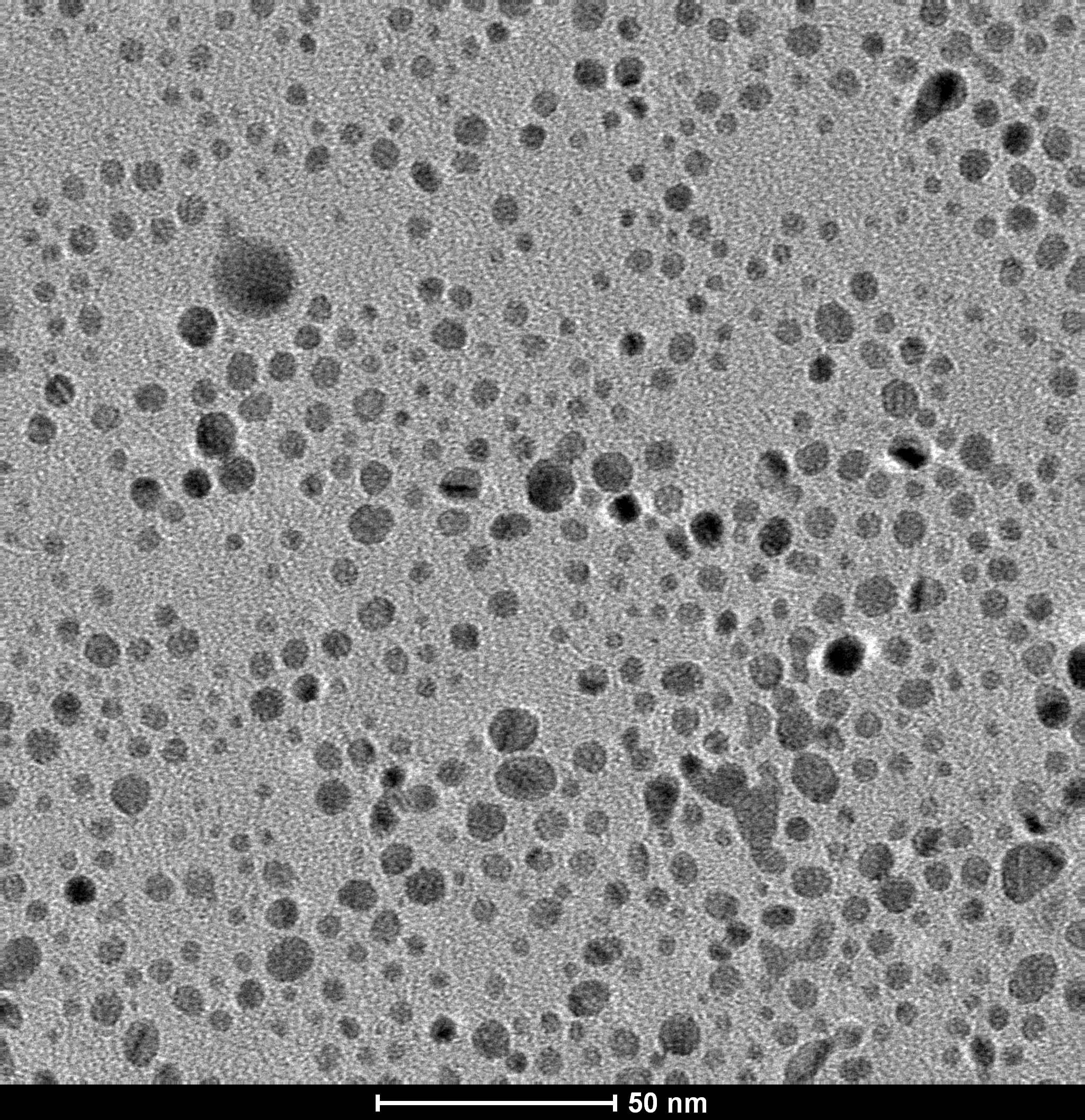

Supplement: Figure S1 — TEM images of 6 nm GA-AgNPs (Left) and 21 nm PVP-AgNPs (right) before any incubation. (DOC) [file pone.0047674.s001.doc]
