# Supplementary material for: Effects of Silver Nanoparticle Exposure on Germination and Early Growth of Eleven Wetland Plants
Source: PLoS One. 2012 Oct 16;7(10):e47674. doi: 10.1371/journal.pone.0047674 (PMC3473015; doi:10.1371/journal.pone.0047674)
Supplement: Table S1 — Physico-chemical characterization of the AgNPs before any incubations. (DOC) [file pone.0047674.s002.doc]

Table S1. Physico-chemical characterization of the AgNPs before any incubations.

|  | Coating | Average Size (nm) | zeta-potential (mV) | shape |
| --- | --- | --- | --- | --- |
| PVP-AgNPs | PVP | 21.0±17.0 | -22.5±1.4 | spherical |
| GA-AgNPs | GA | 6.0±1.7 | -49.5±1.5 | spherical |
