# Supplementary material for: Effects of Silver Nanoparticle Exposure on Germination and Early Growth of Eleven Wetland Plants
Source: PLoS One. 2012 Oct 16;7(10):e47674. doi: 10.1371/journal.pone.0047674 (PMC3473015; doi:10.1371/journal.pone.0047674)
Supplement: Table S2 — Effect of AgNPs and AgNO3 on the seed germination rate (%) of 11 species of wetland plants after 20 days of exposure. (DOC) [file pone.0047674.s003.doc]

Table S2. Effect of AgNPs and AgNO_3_ on the seed germination rate (%) of 11 species of wetland plants after 20 days of exposure.

| **Species** | **DI water** | **PVP-AgNPs (mgAg/L)** | | | **GA-AgNPs (mgAg/L)** | | | **AgNO_3_ (mgAg/L)** | | |
| --- | --- | --- | --- | --- | --- | --- | --- | --- | --- | --- |
|  | 0 | 1 | 10 | 40 | 1 | 10 | 40 | 1 | 10 | 40 |
| *Carex lurida* | 51±10a | 43±6a | 49±10a | 43±8a | 55±6a | 41±8a | 53±12a | 48±4a | 51±11a | 48±4a |
| *Carex crinita* | 49±12a | 48±14a | 46±9a | 47±8a | 51±6a | 45±13a | 41±8a | 43±16a | 52±11a | 58±4a |
| *Carex scoparia* | 72±7a | 71±8a | 75±10a | 72±10a | 77±8a | 76±10a | 65±7a | 77±6a | 75±6a | 87±2b |
| *Carex vulpinoidea* | 58±6a | 45±6a | 44±7a | 48±8a | 43±16a | 52±10a | 41±12a | 37±10a | 53±12a | 60±4a |
| *Scirpus syperinus* | 65±10a | 53±15a | 44±8a | 70±10a | 45±12a | 61±14a | 19±10b | 45±8a | 67±13a | 71±10a |
| *Juncus effusus* | 22±4a | 32±3a | 19±5a | 26±5a | 16±2a | 9±1b | 4±1b | 27±7a | 50±2c | 75±18c |
| *Lolium multiflorum* | 74±9a | 78±5a | 69±13a | 67±8a | 68±14a | 63±11a | 61±9a | 66±16a | 69±7a | 60±12a |
| *Panicum virgatum* | 33±5a | 25±6a | 39±9a | 41±2a | 40±10a | 33±5a | 31±8a | 49±10a | 45±2a | 59±6b |
| *Eupatorium fistulosum* | 52±10a | 66±12a | 64±12a | 64±4a | 67±2a | 82±2b | 79±5b | 67±10a | 85±6b | 88±10b |
| *Lobelia cardinalis* | 8±2a | 8±1a | 9±3a | 6±4a | 5±1a | 8±4a | 5±1a | 8±1a | 5±2a | 5±1a |
| *Phytolacca americana* | 60±8a | 53±16a | 69±6a | 61±8a | 47±8a | 61±2a | 43±8b | 64±10a | 69±5a | 78±6c |

Different letters show significant differences (p < 0.05).
