# Supplementary material for: Effects of Silver Nanoparticle Exposure on Germination and Early Growth of Eleven Wetland Plants
Source: PLoS One. 2012 Oct 16;7(10):e47674. doi: 10.1371/journal.pone.0047674 (PMC3473015; doi:10.1371/journal.pone.0047674)
Supplement: Table S3 — Effect of AgNPs and AgNO3 on the leaf length (cm) of 11 species of wetland plants after 20 days of exposure. (DOC) [file pone.0047674.s004.doc]

Table S3. Effect of AgNPs and AgNO_3_ on the leaf length (cm) of 11 species of wetland plants after 20 days of exposure.

| **Species** | **DI water** | **PVP-AgNPs (mgAg/L)** | | | **GA-AgNPs (mgAg/L)** | | | **AgNO_3_ (mgAg/L)** | | |
| --- | --- | --- | --- | --- | --- | --- | --- | --- | --- | --- |
|  | 0 | 1 | 10 | 40 | 1 | 10 | 40 | 1 | 10 | 40 |
| *Carex lurida* | 1.68±0.20a | 1.50±0.15a | 1.64±0.05a | 2.25±0.16b | 1.99±0.06b | 1.52±0.07a | 1.31±0.11c | 1.66±0.29a | 2.32±0.35b | 1.83±0.09a |
| *Carex crinita* | 1.96±0.15a | 2.06±0.18a | 1.87±0.18a | 2.05±0.09a | 1.95±0.13a | 1.80±0.27a | 1.29±0.30b | 1.53±0.36a | 1.51±0.46a | 1.39±0.05b |
| *Carex scoparia* | 1.31±0.09a | 1.14±0.12a | 1.13±0.15a | 1.26±0.15a | 1.31±0.11a | 1.23±0.15a | 0.90±0.10b | 1.30±0.10a | 1.19±0.11a | 1.24±0.16a |
| *Carex vulpinoidea* | 1.58±0.13a | 1.51±0.17a | 1.55±0.19a | 1.39±0.09a | 1.50±0.31a | 1.47±0.19a | 1.21±0.10b | 1.59±0.21a | 1.37±0.14a | 1.27±0.17b |
| *Scirpus syperinus* | 1.05±0.05a | 1.06±0.08a | 1.03±0.10a | 1.96±0.14a | 0.89±0.05a | 0.82±0.03b | 0.82±0.06b | 1.09±0.10a | 1.04±0.06a | 0.97±0.07a |
| *Juncus effusus* | 0.42±0.08a | 0.47±0.08a | 0.43±0.06a | 0.39±0.13a | 0.41±0.02a | 0.40±0.08a | 0.36±0.04a | 0.44±0.07a | 0.39±0.11a | 0.37±0.18a |
| *Lolium multiflorum* | 5.43±0.41a | 5.47±0.58a | 4.99±0.61a | 4.06±0.25b | 5.23±0.34a | 3.98±0.38b | 2.42±0.35c | 4.89±0.65a | 5.06±0.71a | 3.01±0.28d |
| *Panicum virgatum* | 1.54±0.13a | 1.72±0.06a | 1.48±0.03a | 1.46±0.14a | 1.52±0.16a | 1.51±0.08a | 1.49±0.10a | 1.51±0.08a | 1.47±0.06a | 1.52±0.13a |
| *Eupatorium fistulosum* | 0.40±0.10a | 0.37±0.06a | 0.33±0.06a | 0.38±0.13a | 0.39±0.02a | 0.38±0.02a | 0.31±0.04a | 0.42±0.07a | 0.37±0.11a | 0.30±0.10a |
| *Lobelia cardinalis* | 0.54±0.10a | 0.48±0.06a | 0.50±0.08a | 0.48±0.13a | 0.49±0.04a | 0.48±0.06a | 0.42±0.11a | 0.52±0.07a | 0.47±0.12a | 0.42±0.13a |
| *Phytolacca americana* | 1.43±0.14a | 1.33±0.31a | 1.48±0.12a | 1.29±0.09a | 1.52±0.36a | 1.38±0.18a | 1.53±0.34a | 1.35±0.10a | 1.25±0.14a | 1.42±0.21a |

Different letters show significant differences (p < 0.05).
