# Supplementary material for: Effects of Silver Nanoparticle Exposure on Germination and Early Growth of Eleven Wetland Plants
Source: PLoS One. 2012 Oct 16;7(10):e47674. doi: 10.1371/journal.pone.0047674 (PMC3473015; doi:10.1371/journal.pone.0047674)
Supplement: Table S4 — Effect of AgNPs and AgNO3 on the root length (cm) of 11 species of wetland plants after 20 days of exposure. (DOC) [file pone.0047674.s005.doc]

Table S4. Effect of AgNPs and AgNO_3_ on the root length (cm) of 11 species of wetland plants after 20 days of exposure.

| **Species** | **DI water** | **PVP-AgNPs (mgAg/L)** | | | **GA-AgNPs (mgAg/L)** | | | **AgNO_3_ (mgAg/L)** | | |
| --- | --- | --- | --- | --- | --- | --- | --- | --- | --- | --- |
|  | 0 | 1 | 10 | 40 | 1 | 10 | 40 | 1 | 10 | 40 |
| *Carex lurida* | 3.42±0.51a | 2.38±0.97a | 2.97±0.56a | 5.03±1.11b | 3.11±0.59a | 1.39±0.55c | 0.37±0.08c | 3.40±0.38a | 3.98±0.76a | 1.25±0.25c |
| *Carex crinita* | 4.69±0.77a | 4.02±1.09a | 4.15±0.39a | 3.24±0.06b | 2.51±0.10b | 1.73±0.27c | 0.84±0.13d | 1.56±0.66c | 2.59±0.34b | 0.23±0.04d |
| *Carex scoparia* | 4.53±0.29a | 4.46±0.35a | 3.46±0.35b | 2.53±0.25b | 2.79±0.10bc | 0.53±0.15d | 0.20±0.10d | 4.26±0.31a | 2.06±0.31b | 1.10±0.26d |
| *Carex vulpinoidea* | 4.51±0.26a | 4.47±0.22a | 4.08±0.31a | 1.86±0.15b | 1.99±0.26b | 0.56±0.04c | 0.38±0.08c | 4.44±0.32a | 2.01±0.28b | 1.30±0.17c |
| *Scirpus syperinus* | 0.97±0.14a | 0.91±0.08a | 0.72±0.03a | 0.52±0.05b | 0.71±0.11a | 0.20±0.06c | 0.07±0.06c | 0.77±0.07a | 0.57±0.09b | 0.13±0.06c |
| *Juncus effusus* | 0 | 0 | 0 | 0 | 0 | 0 | 0 | 0 | 0 | 0 |
| *Lolium multiflorum* | 7.27±0.52a | 7.31±0.85a | 6.92±0.51a | 4.89±0.83b | 7.02±0.52a | 3.72±0.41b | 0.75±0.08c | 7.19±0.89a | 6.39±0.74a | 4.12±0.71b |
| *Panicum virgatum* | 1.23±0.06a | 1.28±0.11a | 1.56±0.06a | 2.02±0.14b | 1.84±0.13a | 2.25±0.12b | 0.22±0.07c | 1.56±0.06a | 1.87±0.09a | 2.02±0.11b |
| *Eupatorium fistulosum* | 3.93±0.12a | 3.86±0.15a | 3.69±0.10a | 3.39±0.21a | 3.79±0.20a | 0.80±0.20b | 0.51±0.10b | 3.83±0.15a | 3.43±0.35a | 0.90±0.10b |
| *Lobelia cardinalis* | 1.50±0.11a | 1.46±0.06a | 1.48±0.14a | 0.80±0.14b | 1.56±0.16a | 1.44±0.08a | 0.58±0.09b | 1.39±0.21a | 1.52±0.18a | 0.62±0.08b |
| *Phytolacca americana* | 1.68±0.23a | 1.40±0.72a | 2.81±0.41ac | 4.63±0.12bcd | 2.04±0.06a | 6.37±0.23d | 2.01±0.53a | 2.17±0.60ac | 4.68±0.22bcd | 5.97±0.11d |

Note: Different letters show significant differences (p < 0.05).
